# Cognitive performance in irritable bowel syndrome: evidence of a stress-related impairment in visuospatial memory

**DOI:** 10.1017/S0033291713002171

**Published:** 2013-08-29

**Authors:** P. J. Kennedy, G. Clarke, A. O‘Neill, J. A. Groeger, E. M. M. Quigley, F. Shanahan, J. F. Cryan, T. G. Dinan

**Affiliations:** 1 Alimentary Pharmabiotic Centre, University College Cork, Ireland; 2 Department of Psychiatry, University College Cork, Ireland; 3 Department of Psychology, University of Hull, UK; 4 Department of Medicine, University College Cork, Ireland; 5 Department of Anatomy and Neuroscience, University College Cork, Ireland

**Keywords:** CANTAB, cognition, cortisol, irritable bowel syndrome (IBS), stress

## Abstract

**Background:**

Central nervous system (CNS) dysfunction is a prominent feature of the functional gastrointestinal (GI) disorder, irritable bowel syndrome (IBS). However, the neurobiological and cognitive consequences of key pathophysiological features of IBS, such as stress-induced changes in hypothalamic–pituitary–adrenal (HPA)-axis functioning, is unknown. Our aim was to determine whether IBS is associated with cognitive impairment, independently of psychiatric co-morbidity, and whether cognitive performance is related to HPA-axis function.

**Method:**

A cross-sectional sample of 39 patients with IBS, a disease control group of 18 patients with Crohn's disease (CD) in clinical remission and 40 healthy age- and IQ-matched control participants were assessed using the Paired Associates Learning (PAL), Intra-Extra Dimensional Set Shift (IED) and Spatial Working Memory (SWM) tests from the Cambridge Neuropsychological Test Automated Battery (CANTAB) and a computerized Stroop test. HPA-axis function was determined by measuring the cortisol awakening response (CAR).

**Results:**

IBS patients exhibited a subtle visuospatial memory deficit at the PAL six- pattern stage (*p* = 0.03), which remained after psychiatric co-morbidity was controlled for (*p* = 0.04). Morning cortisol levels were lower in IBS (*p* = 0.04) and significantly associated with visuospatial memory performance within IBS only (*p* = 0.02).

**Conclusions:**

For the first time, altered cognitive function on a hippocampal-mediated test of visuospatial memory, which was related to cortisol levels and independent of psychiatric co-morbidity, has been identified in IBS. Visuospatial memory impairment may be a common, but currently neglected, component of IBS. Further elucidation of the nature of this impairment may lead to a greater understanding of the underlying pathophysiology of IBS, and may provide novel therapeutic approaches.

## Introduction

Irritable bowel syndrome (IBS) is the most common functional gastrointestinal (GI) disorder in Western countries, affecting an estimated 10% of the general population (Clarke *et al.* 2009 *b*) and accounting for up to 50% of all visits to general practitioners for GI complaints (Wilson *et al.* 2004). Symptoms, which include abdominal pain or discomfort and altered bowel habits (Longstreth *et al.* 2006), are chronic (Ford *et al.* 2008) in some cases debilitating, and are cited as a leading cause of work absenteeism and presenteeism (Spiegel, 2009). IBS is thus associated with a substantial impairment in the individual sufferer's quality of life (Longstreth *et al.* 2003).

Although the pathophysiology of IBS is not fully understood, it is best regarded as a disorder caused by dysregulation of the complex interactions along the brain–gut axis (Cryan & O'Mahony, 2011; Grenham *et al.* 2011; Mayer & Tillisch, 2011; Collins *et al.* 2012), a model describing the bidirectional communication between the central, autonomic and enteric nervous systems, involving neural, endocrine and immune pathways (Ohman & Simren, 2007). For example, it is thought that stress-induced changes in hypothalamic–pituitary–adrenal (HPA)-axis functioning can dysregulate normal brain–gut interactions (Mayer, 2000) and studies from our laboratory and others have documented abnormal HPA-axis activity in IBS (Bohmelt *et al.* 2005; Dinan *et al.* 2006; Chang *et al.* 2009). A role for low-grade immune activity in IBS has garnered much support from experimental studies demonstrating alterations on several immune parameters (Barbara *et al*. 2004; Collins, 2005; Guilarte *et al.* 2007; Ohman & Simren, 2010), including elevated peripheral levels of pro-inflammatory cytokines (Dinan *et al.* 2006, 2008; Liebregts *et al.* 2007; Scully *et al.* 2010; McKernan *et al.* 2011) and an immune-mediated degradation of tryptophan metabolism along the kynurenine pathway (Fitzgerald *et al.* 2008; Clarke *et al.* 2009 *a*, 2012). Moreover, central dysfunction is a prominent feature of IBS. Centrally acting therapies, both psychotherapeutic (Whorwell *et al*. 1984, 1987; Lackner *et al.* 2006) and pharmacological (Creed *et al.* 2003), have demonstrated efficacy in the treatment of GI symptoms in IBS. Studies commonly report an increased prevalence of depression and anxiety, and affected patients often have a formal, psychiatrically diagnosed mood disorder (Hazlett-Stevens *et al.* 2003; Creed *et al.* 2005, 2008; Lembo *et al.* 2009; Mykletun *et al.* 2010). In addition, mounting evidence from functional magnetic resonance imaging (fMRI) research indicates that, in response to visceral pain stimulation, patients with IBS exhibit abnormal brain activity in regions involved in pain processing and endogenous pain modulation (Mayer *et al.* 2009; Tillisch *et al.* 2011). However, a largely understudied and potentially underestimated manifestation of brain–gut axis dysfunction in IBS is cognitive impairment (Kennedy *et al.* 2012).

To date, the few studies assessing cognitive performance in IBS are in line with a cognitive behavioural framework (Sharpe *et al.* 1992; Creed, 2007; Spence & Moss-Morris, 2007) by demonstrating that patients exhibit attentional biases to GI symptom-related stimuli (Gibbs-Gallagher *et al.* 2001; Afzal *et al.* 2006; Posserud *et al.* 2009; Martin & Chapman, 2010; Chapman & Martin, 2011) or negatively valenced words (Gomborone *et al.* 1993). However, we recently proposed a cognitive neurobiological model whereby some of the key pathophysiological features of IBS, including stress, immune activation and chronic pain, may impact not only on emotion- or symptom-related cognition but also on key domains of executive function, working memory, attention and episodic memory (Kennedy *et al.* 2012). This model has received support from an fMRI study reporting impaired cognitive flexibility on the Wisconsin Card Sorting Test (WCST), with associated differences in regional activity in frontal and temporal brain regions in IBS (Aizawa *et al.* 2012). Although this is an important and substantial step in elucidating the cognitive domains that are affected in IBS, it is limited in its scope of assessment by focusing on executive function alone. As such, the impact on other key brain functions such as episodic and working memory or non-emotional/symptom-related attention in IBS remains unknown. Furthermore, there remains a lack of understanding of how key pathophysiological features of IBS, such as stress-induced changes in HPA-axis functioning (Dinan *et al.* 2006), can impact on the brain and lead to cognitive impairment. Considering the prevalence of IBS and the high incidence among young adult females who may be engaged in formal education or embarking on careers, it is of great importance to explore the extent of cognitive impairment in this debilitating functional GI disorder.

The current study aimed to expand on previous findings by assessing how IBS patients perform on a battery of cognitive tests covering cognitive flexibility and attentional set-shifting (Intra-Extra Dimensional Set Shift; IED), working memory (Spatial Working Memory; SMW) and visuospatial episodic memory (Paired Associates Learning; PAL) from the Cambridge Neuropsychological Test Automated Battery (CANTAB®), and response conflict using the Stroop test. Considering that IBS is a stress-related disorder, our primary hypothesis was that, in addition to impaired cognitive flexibility as described previously (Aizawa *et al.* 2012), patients with IBS would exhibit mild but identifiable hippocampal-mediated, visuospatial memory dysfunction, which would be related to HPA-axis functioning as measured by the cortisol awakening response (CAR). In addition, we included the SWM and Stroop tests based on the hypothesis that impaired working memory and inhibitory responding have been identified in other conditions associated with stress and chronic pain, such as fybromyalgia and chronic fatigue syndrome (Glass, 2006; Moriarty *et al.* 2011), and thus performance on these tests may also be compromised in IBS. We compared cognitive performance in IBS with that of a group of individuals with a well-defined inflammatory GI disorder, Crohn's disease (CD). These patients were included as our disease control group to control for the effects of chronic GI symptoms. Additionally, we explicitly controlled for any possible impact of anxiety and depression on cognitive performance by carrying out a reanalysis of the data where cognitive differences were identified, following the removal of participants exhibiting clinically relevant levels of psychiatric co-morbidity.

## Method

### Participants

Patients with IBS who satisfied Rome III criteria (Longstreth *et al.* 2006) and had undergone previous investigation to exclude the presence of organic GI disease, including inflammatory bowel disease (IBD) and coeliac disease, and patients with CD in clinical remission [Harvey Bradshaw Index (HBI) score < 5] were recruited from speciality clinics at Cork University Hospital. Healthy control participants were recruited from the staff and student population of University College Cork by advertisement. Patients presenting with significant acute or chronic coexisting illness other than that under study were excluded. Study participants were males and females between 18 and 50 years of age. Exclusion criteria included use of psychoactive medications (anxiolytics, antipsychotics, antidepressants at a dose used to treat depressive disorders, and opioid-based pain relievers), regular use of non-steroidal anti-inflammatory drugs (NSAIDs), antibiotic use within the prior 4 weeks, history of alcohol abuse, evidence of immunodeficiency and recent (within 6 months) abdominal surgery. Patients with CD were required to have at least a 1-month wash-out period from prednisone use prior to testing. Budesonide, which has minimal systemic bioavailability, was allowed. Healthy control participants were excluded if they had a history of chronic complaints, GI (not attributable to gastroenteritis or other clearly identifiable cause) or otherwise.

### Study procedures

The study protocol (APC024 2010) and all procedures were approved by the University College Cork Clinical Research Ethics Committee of the Cork Teaching Hospitals and conducted in accordance with the International Conference on Harmonization (ICH) Good Clinical Practice (GCP) Guidelines and the Declaration of Helsinki. Study participants meeting inclusion criteria for entry provided written informed consent before participation. A total of 97 individuals enrolled in the study. Groups were matched on the basis of age, verbal IQ, body mass index (BMI) and units of alcohol consumed per week. Study visits were conducted between 0730 and 1030 h to control for endogenous fluctuations in glucocorticoid levels. On arrival at the laboratory, a brief medical examination was carried out by an experienced clinical research nurse who measured participants' vital signs, recorded their BMI and collected a venous blood sample for assessment of full blood count, renal function, serum electrolytes and liver enzymes. Clinically significant abnormalities in these blood tests led to exclusion from the study following review by an experienced physician.

### Measures

#### Mood and GI symptom questionnaires

Symptoms of anxiety and depression were assessed using the self-reported Hospital Anxiety and Depression Scale (HADS; Zigmond & Snaith, 1983) and the nine-item Patient Health Questionnaire (PHQ-9; Kroenke *et al.* 2001). Patients with IBS rated their current abdominal pain severity on a scale from 0 (no pain) to 100 (severe pain). CD patients completed the HBI as a general index of current symptomatology.

#### Salivary cortisol analysis

HPA-axis functioning was examined by measuring the CAR. This is a well-validated method for characterizing HPA-axis function and has been used for this purpose in numerous functional and psychiatric disorders (Fries *et al.* 2009). On the morning prior to their study visit, participants were instructed to collect saliva samples upon wakening, and at 1 h and 3 h post-wakening. As it has not yet been established whether variable waking times affect the CAR (for reviews, see Fries *et al.* 2009; Law *et al.* 2013), we took the approach that is common in the literature (Hinkelmann *et al.* 2013) and did not require that participants wake at a specific time, but followed their normal routine as closely as possible. Waking times were recorded for analysis to determine any group differences. Salivary samples were stored at –80°C until analysis. Cortisol concentrations were determined using the Cortisol Enzyme Immunoassay Kit according to the manufacturer's instruction (Enzo® Life Sciences, UK). The assay detection limit was 0.16 nmol/l. Inter- and intra-assay coefficients of variation (CVs) were 11.24% and 8.2% respectively.

### Cognitive assessment

Tests from the CANTAB® battery (Cambridge Cognition, Ltd, UK; Robbins & Sahakian, 1994) and a computerized Stroop word–colour interference test (Stroop; Xavier Educational Software Ltd, UK) were administered by a trained test administrator who issued standardized verbal instructions to participants on the use of a portable touch screen Sahara i440D Slate Tablet PC (TabletKiosk, Sand Dune Ventures, USA) running CANTABeclipse™ software. The cognitive assessment lasted approximately 45 min with each participant first completing the Big/Little Circle as a short familiarization task, followed by the IED, PAL and SWM tests from the CANTAB, and finally the Stroop test. A measure of pre-morbid IQ was obtained using the National Adult Reading Test-2 (NART-2; Nelson & Willison, 1991) and converted to Wechsler Adult Intelligence Scale – Revised (WAIS-R) full-scale IQ scores. A brief description of each cognitive test is provided in the following sections. More detailed information on CANTAB tests is available elsewhere (Sahakian & Owen, 1992; Fray & Robbins, 1996).

#### PAL, parallel mode

PAL is a test of visuospatial episodic memory and assesses new learning, list memory and list learning, and has demonstrated sensitivity to changes in the function of hippocampal brain regions (Owen *et al.* 1995; Sweeney *et al.* 2000; Swainson *et al.* 2001; Blackwell *et al.* 2004). However, performance on PAL has also been shown to engage additional brain regions comprising the frontoparietal network during encoding phases, and posterior cingulate and left cuneus regions during retrieval stages (de Rover *et al.* 2011). Outcome measures assessed were errors made at the six-pattern and eight-pattern stage (adjusted), total errors (adjusted), mean trials to success, and first trial memory score.

#### Stroop test

The Stroop is a test of executive function and assesses selective attention and response inhibition. Inhibition of the prepotent response in the interference stage of the Stroop primarily engages the anterior cingulate cortex (ACC), with general Stroop performance also requiring input from regions of the temporal and parietal lobes (Botvinick *et al.* 2004; Alvarez & Emory, 2006; Strauss *et al.* 2006). The computerized Stroop test used in the current study is based on the Victoria Stroop Test (VST) as described previously (Assef *et al.* 2007). Response speed in milliseconds is recorded on each trial with an overall mean response time (MRT) for each of three stages [Stage 1, word naming; Stage 2, colour naming; Stage 3 (interference stage), naming the colour of an incongruent word, for example the word ‘blue’ printed in the colour red], consisting of 24 trials. The main outcome measure is the ‘Stroop effect’, calculated by subtracting the MRT of Stage 1 from the MRT on Stage 3 (Assef *et al.* 2007).

#### IED

IED is a test of executive function and assesses rule acquisition and reversal, attentional set formation, maintenance and shifting (Downes *et al.* 1989; Sahakian & Owen, 1992). Reversal learning has been shown to involve the ventral prefrontal cortex (PFC) whereas the dorsolateral PFC is engaged during attentional set-shifting (Nagahama *et al.* 2001). The outcome measures assessed were reversal learning (errors made on stages 2, 5, 7 and 9) and attentional flexibility (errors made on stages 6 and 8), as described previously (Mehta *et al.* 1999; Rahman *et al.* 1999), and total errors (adjusted).

#### SWM

SWM is a test of working memory, involving online monitoring and updating of information and self-ordered searching, and has shown sensitivity to frontal lobe dysfunction (Owen *et al.* 1996; Robbins *et al.* 1998). The outcome measures assessed were total between-search errors, total errors and strategy score.

### Statistical analysis

As the CANTAB variables were not normally distributed, the following transformations were applied. IED and PAL outcome variables were normalized using log_10_ transformations and SWM outcome variables were normalized using square-root transformations. The Stroop was completed by 38 controls, 38 IBS patients and 18 CD patients, and data were analysed excluding the three participants who did not complete the test. The *χ*^2^ test was used to examine the gender distribution across groups. A one-way ANOVA was used to explore differences in participant waking times, group characteristics (age, IQ, BMI, units of alcohol per week), HADS-anxiety (HADS-A), HADS-depression (HADS-D) and PHQ-9 scores, and to assess individual measures of the PAL, IED, SWM and Stroop tests, followed by Tukey's Honestly Significant Difference (HSD) *post-hoc* test or Dunnett's *t* contrasts, as appropriate. As this was an exploratory study, we have not specified a single study end-point and did not adjust for multiple end-points in a full factorial model (Bender & Lange, 2001; Feise, 2002) to allow detection of possible differences in cognitive performance on each test within our study population. Total cortisol levels across each collection time point were determined using an area under the curve with respect to ground (AUC_g_) calculation (Pruessner *et al.* 2003). AUC_g_ cortisol levels were transformed using a natural log (ln) to correct positive skew and improve homogeneity of variance, followed by a one-way ANOVA and Dunnett's *t* contrasts to identify patient *versus* control differences. Saliva samples were provided by 32 healthy controls, 36 IBS patients and 17 CD patients as instructed and useable for analysis. Correlation analyses were carried out using Spearman's *ρ* within each group (control/IBS/CD) to examine the influence of total morning cortisol levels (AUC_g_), IQ, anxiety (HADS-A), depression (HADS-D; PHQ-9), duration of disease, current abdominal pain severity in patients with IBS, and HBI total scores in CD patients on cognitive performance. Finally, as an additional measure to control for the influence of levels of anxiety or depression on cognitive performance, participants meeting predefined criteria for possible co-morbid depression (a PHQ-9 score of ⩾10; Kroenke *et al.* 2001) and/or co-morbid anxiety (a HADS-A score of ⩾11; Snaith, 2003) were excluded, and a one-way ANOVA along with Tukey comparisons and Dunnett's *t* contrasts was carried out on CANTAB and Stroop outcomes, where group differences had been identified with all participants included. Sample size was determined using a power calculation to detect significant group differences at an *α* level of 0.05. The study was not powered to detect subgroup differences among IBS patients, although this information was recorded. Non-transformed data are presented as mean ± standard error of the mean (s.e.m.). Effect sizes are reported as partial eta squared (*η*_p_^2^). All statistical procedures were carried out using SPSS version 20.0 (SPSS Inc., USA).

## Results

### Sample characteristics

Group characteristics for healthy control participants, IBS patients and CD patients are presented in Table 1. Groups did not differ significantly on age, IQ, BMI or number of units of alcohol consumed per week. One patient with CD was using budesonide (Entocort EC) at the time of testing. According to Rome III criteria, seven IBS patients were diarrhoea predominant (IBS-D), four constipation predominant (IBS-C) and 28 were mixed (IBS-M). Patients with IBS (*p <* 0.001) and patients with CD (*p* = 0.02) scored higher on the HADS-D than the healthy controls. Patients with IBS scored significantly higher on the PHQ-9 depression scale (*p <* 0.001) and the HADS-A (*p <* 0.001) than the healthy controls.

**Table 1.** Comparison of group demographics and clinical characteristics

	Healthy controls (*n* = 40)	IBS (*n* = 39)	CD (*n* = 18)	*p* value
Age (years)	28.3 ± 1.43	28.21 ± 1.19	32.67 ± 2.11	0.14
Gender				
Male	11 (27.5)	6 (15.4)	11 (61.1)	
Female	29 (72.5)	33 (84.6)	7 (38.9)	0.002
BMI (kg/m^2^)	23.55 ± 0.59	23.74 ± 0.63	25.23 ± 0.73	0.23
WAIS-R full-scale IQ (NART conversion)	108.57 ± 1.07	105.48 ± 1.39	103.39 ± 2.54	0.07
HADS-D	0.85 ± 0.15	3.85 ± 0.46	2.99 ± 0.70	<0.001
HADS-A	4.30 ± 0.4	8.21 ± 0.74	6.67 ± 3.45	<0.001
PHQ-9	2.03 ± 0.36	6.1 ± 0.86	3.89 ± 0.94	<0.001
Disease duration (years)	–	9.13 ± 1.13	9.72 ± 1.63	
Current pain, abdominal pain severity (0–100)	–	34.75 ± 4.65	–	
HBI total score (remission < 5)	–	–	1.89 ± 0.31	

IBS, Irritable bowel syndrome; CD, Crohn's disease; BMI, body mass index; WAIS-R, Wechsler Adult Intelligence Scale – Revised; NART, National Adult Reading Test; HADS-A/D, Hospital Anxiety and Depression Scale – Anxiety/ Depression; PHQ-9, nine-item Patient Health Questionnaire; HBI, Harvey Bradshaw Index.

Data are given as *n* (%) or mean ± standard error of the mean (s.e.m.).

### Cognitive performance

#### Visuospatial episodic memory (PAL)

**Fig. 1.**
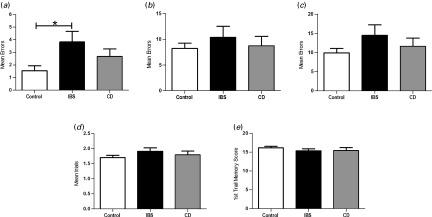
Group comparison of visuospatial episodic memory performance on the Paired Associates Learning (PAL) test. (*a*) Mean errors at the six-pattern stage [* *p* < 0.05, irritable bowel syndrome (IBS) *versus* control]; (*b*) mean errors at the eight-pattern stage; (*c*) mean total errors; (*d*) average trials needed to successfully complete each stage; (*e*) average number of correct choices on the first trial of each stage; lower score = poorer performance. CD, Crohn's disease. Data are presented as mean ± standard error of the mean (s.e.m.).

Comparisons of the number of errors committed at each individual stage showed that the six-pattern stage (*F*_2,94_ = 3.43, *p* = 0.03, *η*_p_^2^ = 0.07; Fig. 1 *a*), but not the eight-pattern stage (*F*_2,94_ = 0.02, *p* = 0.98; Fig. 1 *b*), differentiated patients from healthy controls. *Post-hoc* exploration of this group effect at the six-pattern stage using Tukey comparisons found that patients with IBS exhibited significantly (*p* = 0.03) impaired visuospatial memory performance compared to controls but not to patients with CD (*p* = 0.97). Patients with CD did not differ from controls in the number of errors made at the six-pattern stage (*p* = 0.2). There was no overall effect of group on total errors (*F*_2,94_ = 0.46, *p* = 0.63, *η*_p_^2^ = 0.01; Fig. 1 *c*), mean number of trails to success (*F*_2,94_ = 1.05, *p* = 0.35, *η*_p_^2^ = 0.02; Fig. 1*d*) or first trial memory score (*F*_2,94_ = 1.17, *p* = 0.32, *η*_p_^2^ = 0.02; Fig. 1 *e*).

#### Selective attention and response inhibition (Stroop)

Group differences on the Stroop effect approached significance (*F*_2,91_ = 3.04, *p* = 0.052, *η*_p_^2^ = 0.06). Inspection of *post-hoc* Tukey comparisons showed that patients with CD (*p* = 0.04), but not patients with IBS (*p* = 0.9), were significantly less able to cope with Stroop interference than healthy controls (Table 2).

**Table 2.** Summary of mean test scores on the Stroop, IED and SWM

Cognitive test	Control	IBS	CD
Stroop effect (ms)	297.55 ± 29.22	320.31 ± 38.96	463.89 ± 81.52*
IED			
Stages complete	8.5 ± 0.14	8.5 ± 0.14	8.44 ± 0.2
Total errors (adjusted)	24.67 ± 3.23	24.51 ± 3.25	27.17 ± 4.67
Reversal learning (errors)	5.05 ± 0.44	6.23 ± 0.92	7.67 ± 2.17
Attentional flexibility (errors)	11.92 ± 1.72	10.79 ± 1.54	11.89 ± 2.43
SWM			
Total between errors	18.57 ± 2.81	23.43 ± 2.57	25.44 ± 4.62
Total errors	19.4 ± 3.02	24.15 ± 2.68	26.5 ± 4.81
Strategy score	30.25 ± 1.04	31.9 ± 0.99	29.78 ± 1.63

IED, Intra-Extra Dimensional Set Shift; SWM, Spatial Working Memory; IBS, irritable bowel syndrome; CD, Crohn's disease.

* *p* < 0.05 *v.* control.

Data are given as mean ± standard error of the mean (s.e.m.).

#### Executive function (IED)

Groups did not differ significantly on total stages complete (*F*_2,94_ = 0.04, *p* = 0.9, *η*_p_^2^ = 0.001), measures of reversal learning (*F*_2,94_ = 0.68, *p* = 0.51, *η*_p_^2^ = 0.01), attentional flexibility (*F*_2,94_ = 0.053, *p = 0.*95, *η*_p_^2^ = 0.001) or the total number of errors across all stages of the IED test (*F*_2,94_ = 0.16, *p* = 0.85, *η*_p_^2^ = 0.003; see Table 2 for mean scores).

#### SWM

No significant main effect of group was found for the SWM strategy test (*F*_2,94_ = 0.92, *p* = 0.4, *η*_p_^2^ = 0.02), total between errors (*F*_2,94_ = 1.45; *p* = 0.24, *η*_p_^2^ = 0.03) or total errors across each stage (*F*_2,94_ = 1. 36; *p* = 0.26, *η*_p_^2^ = 0.02, see Table 2 for mean scores).

#### Morning cortisol levels

**Fig. 2.**
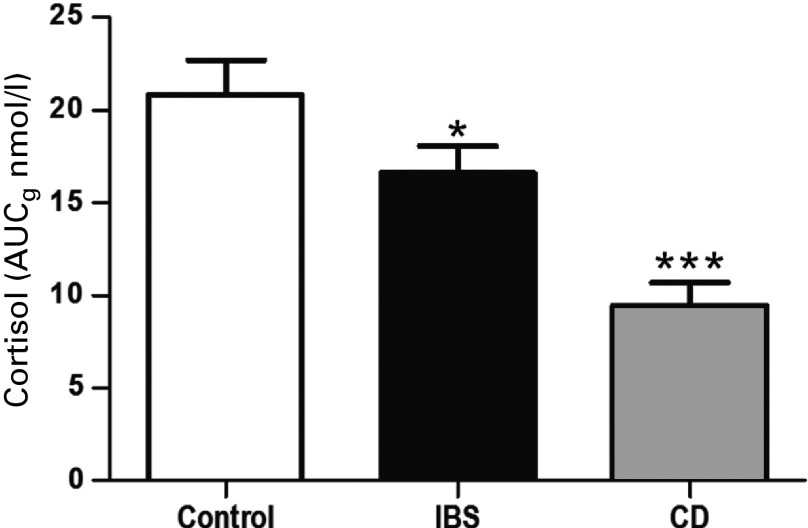
Group comparison of total morning cortisol levels determined using an area under the curve with respect to ground (AUC_g_) calculation on all three measurement points in healthy control participants (*n* = 34), patients with irritable bowel syndrome (IBS; *n* = 36) and patients with Crohn's disease (CD; *n* = 17). * *p* < 0.05, *** *p* < 0.001 *versus* control. Data are presented as mean ± standard error of the mean (s.e.m.).

Waking times for healthy controls (mean = 0813 h ± 13 min), patients with IBS (mean = 0820 h ± 14 min) and patients with CD (mean = 0808 h ± 26 min) were not significantly different (*F*_2,82_ = 0.12, *p* = 0.89). Total morning cortisol levels (AUC_g_) differed significantly between patients and controls (*F*_2,82_ = 13.71, *p <* 0.001, *η*_p_^2^ = 0.25; Fig. 2). *Post-hoc* analysis using Dunnett's *t* contrasts against controls showed that patients with IBS (*p* = 0.03) and patients with CD (*p* < 0.001) exhibited lower total cortisol levels across the three measurement time points in comparison to healthy controls.

### Correlation analysis

A correlation analysis was carried out to identify relationships between visuospatial memory performance (errors at the PAL six-pattern stage), selective attention and response inhibition (the Stroop test), total morning cortisol levels (AUC_g_), IQ (WAIS-R), levels of anxiety (HADS-A) and levels of depression (HADS-D, PHQ-9). A significant negative relationship was identified between AUC_g_ and visuospatial memory performance within the IBS group but not in the healthy control participants or the patients with CD (Table 3). Additional correlations were carried out within the patient groups to examine the relationship between disease duration, current abdominal pain (IBS patients) and HBI (CD patients), and cognitive performance. Within the IBS group, no significant relationships were identified between disease duration or current abdominal pain and PAL six-pattern errors (both *p* > 0.05). Similarly, within the CD group, no significant relationship was identified between disease duration or HBI scores and Stroop performance (both *p* > 0.05).

**Table 3.** Summary of correlations between cognitive performance (errors at the PAL six-pattern stage and the Stroop test) and morning cortisol levels, IQ and clinical characteristics

Measure	Errors PAL six-pattern stage			Stroop test		
	Control	IBS	CD	Control	IBS	CD
Morning cortisol (AUC_g_)	0.023	−0.367*	−0.437	−0.005	−0.013	0.032
WAIS-R IQ	−0.237	−0.121	−0.035	0.080	−0.113	0.266
HADS-A (Anxiety)	0.114	0.008	−0.047	0.130	−0.142	0.317
HADS-D (Depression)	0.005	0.066	0.361	0.237	−0.107	0.351
PHQ-9 (Depression)	−0.013	0.025	−0.038	0.089	0.072	0.284
Disease duration	–	0.18	0.16	–	−0.01	0.37
Current abdominal pain	–	−0.27	–	–	−0.02	–
HBI	–	–	−0.07	–	–	−0.20

PAL, Paired Associate Learning; WAIS-R, Wechsler Adult Intelligence Scale – Revised; HADS-A/D, Hospital Anxiety and Depression Scale – Anxiety/Depression; PHQ-9, nine-item Patient Health Questionnaire; HBI, Harvey Bradshaw Index.

* *p* < 0.05.

### Cognitive performance after controlling for psychiatric co-morbidity

To further control for any influence of levels of depression and anxiety on cognitive performance, participants meeting the predefined criteria for psychiatric co-morbidity (PHQ-9 score of ⩾10; Kroenke *et al.* 2001) and/or co-morbid anxiety (HADS-A score of ⩾11; Snaith, 2003) were excluded and we reanalysed the number of errors made at the PAL six-pattern stage and the performance on the Stroop interference test. The predefined criteria for psychiatric co-morbidity were met by 16 (41.02%) of the IBS patients and five (27.78%) of the CD patients but none of the control participants. Reanalysis using ANOVA showed that group differences on visuospatial episodic memory performance (errors at PAL six-pattern stage; control: *n* = 40, IBS: *n* = 23, CD: *n* = 13) remained significant (*F*_2,73_ = 3.39, *p* = 0.03, *η*_p_^2^ = 0.08). *Post-hoc* analysis using Tukey comparisons showed that patients with IBS and without psychiatric co-morbidity made significantly more errors at the PAL six-pattern stage than healthy controls (*p* = 0.04). Overall, group differences on selective attention and response inhibition (Stroop interference effect; control: *n* = 38, IBS: *n* = 22, CD: *n* = 13), after controlling for psychiatric co-morbidity, did not reach significance (*F*_2,70_ = 2.11; *p* = 0.129, *η*_p_^2^ = 0.57). However, *post-hoc* inspection of Dunnett's *t* contrasts showed that patients with CD without psychiatric co-morbidity, when compared to healthy control participants, still exhibited reduced attentional capacity, as they continued to be significantly less able to manage Stroop interference than controls (*p* = 0.04).

## Discussion

In this study we tested the hypothesis that IBS is associated with cognitive impairment. We used CANTAB, a highly standardized and well-validated cognitive test battery (Strauss *et al.* 2006), to assess performance in IBS and CD in comparison with healthy controls across several cognitive domains including reversal learning and attentional flexibility (IED), selective attention and response inhibition (Stroop), working memory (SWM) and visuospatial episodic memory (PAL). To our knowledge, this is the first time that such a detailed cognitive assessment has been performed in either condition.

In support of our main hypothesis, we have shown that patients with IBS exhibit a subtle deficit in visuospatial episodic memory functioning, which was clear at the six-pattern stage of PAL. Performance on PAL is highly dependent on the functioning of the hippocampus (de Rover *et al.* 2011), a brain region that is intrinsically involved in the negative feedback regulation of the HPA axis and where glucocorticoid receptors are highly expressed (McEwen, 1999). In our sample, patients with IBS exhibited lower basal morning cortisol levels in comparison to healthy controls, a finding that is in support of some (Bohmelt *et al.* 2005; Suarez-Hitz *et al.* 2012), but not all (Chang *et al.* 2009), previous reports. Nonetheless, these results add to the well-acknowledged view that HPA-axis dysfunction is a key feature of IBS (Mayer *et al.* 2001). Furthermore, our finding of a relationship between morning cortisol levels and performance on the PAL test is in line with many preclinical (Song *et al.* 2006; Sterlemann *et al.* 2010) and human studies (Lupien *et al.* 2009) that have documented the negative impact of HPA-axis dysregulation on hippocampal-mediated cognitive performance. Our results suggest that lower cortisol levels are associated with increased PAL errors in IBS, and this is in line with the commonly acknowledged inverted U-shape function of cortisol on hippocampal-mediated memory performance, where both abnormally elevated and blunted cortisol levels are associated with cognitive dysfunction (de Kloet *et al.* 1999; Wolf, 2003; Lupien *et al.* 2007). Indeed, previous cognitive assessments in IBS (Gomborone *et al.* 1993; Gibbs-Gallagher *et al.* 2001; Afzal *et al.* 2006; Posserud *et al.* 2009; Martin & Chapman, 2010; Chapman & Martin, 2011), together with our findings, suggest that IBS may be associated with both emotionally modulated cognitive alterations mediated by amygdalar regions and non-emotional visuospatial episodic memory alterations mediated by the hippocampus.

Brain imaging studies have suggested a key role for the ACC in abnormal central processing of visceral pain in IBS (Mayer *et al.* 2009). Patients with IBS in our sample did not exhibit deficits on the Stroop test, which heavily engages the ACC during the interference stages (Alvarez & Emory, 2006), suggesting that the ACC is unaffected at the cognitive level and that abnormal functioning of this region in IBS is specific to altered visceral pain processing. However, subregions of the ACC (the perigenual ACC and anterior midcingulate cortex) may be functioning differentially in IBS (Tillisch *et al.* 2011) and our findings may simply reflect that the Stroop does not fully engage these particular regions. Furthermore, it should be noted that altered visceral pain processing may be apparent in only a subset of patients with IBS (Quigley, 2006) and, as such, it is possible that only a subset of patients, not represented in our sample, may exhibit ACC-related cognitive deficits.

Altered cognitive flexibility on the WCST, accompanied by reduced right dorsolateral PFC activity, has recently been described in IBS (Aizawa *et al.* 2012). By contrast, we found no deficit in reversal learning or cognitive flexibility on the IED, which is considered to be an analogue of the WCST (Robbins *et al.* 1998). Furthermore, the SWM and IED tasks engage the mid-dorsolateral/ventral PFC and the dorsolateral PFC respectively (Owen *et al.* 1995; Nagahama *et al.* 2001). As no group differences were identified on either of these tests, we found no evidence to support a general decrement in dorsolateral PFC-mediated cognitive function. Explanations for these disparate results may include the heterogeneity of IBS, differences in central functioning between IBS subtypes, temporal symptom fluctuations or subtle differences in the cognitive requirements for adequate performance on the IED and the WCST. Therefore, future studies exploring other aspects of executive function coupled with functional brain imaging are needed to clarify the impact, if any, on executive processes and other cognitive functions mediated by frontal brain regions in IBS.

In accordance with previous reports, levels of both depression and anxiety were higher in IBS (Hazlett-Stevens *et al.* 2003; Creed *et al.* 2005, 2008; Lembo *et al.* 2009; Mykletun *et al.* 2010) and depression was slightly elevated in CD (Goodhand *et al.* 2012). Cognitive deficits are well documented in depressive and anxiety disorders (Austin *et al.* 2001) and a relationship between the CAR and hippocampal-mediated cognition in unmedicated depressed patients has been described recently (Hinkelmann *et al.* 2013). It is important to note that the cognitive deficits most clearly exhibited by patients with IBS (visuospatial episodic memory; PAL) and by patients with CD (Stroop interference) persisted after removal of patients exhibiting possible psychiatric co-morbidity, which strongly suggests that these deficits are independent of psychiatric co-morbidity. This result further suggests that a shared underlying pathophysiological mechanism, such has heightened levels of pro-inflammatory cytokines, which have been identified in both IBS (Dinan *et al.* 2006; Liebregts *et al.* 2007; Dinan *et al.* 2008; Scully *et al.* 2010; McKernan *et al.* 2011) and depression (Raison *et al.* 2006) and can impact on cognitive performance (Kennedy *et al.* 2012), may play a pivotal role in mediating the similarities in cognitive deficits identified in each disorder. Moreover, it is likely that similarities in cognitive dysfunction described here in IBS not only apply to depression but also overlap with other functional or psychiatric disorders in which stress, immune activation or altered central pain processing is implicated in the underlying pathophysiology. However, a note of caution is warranted in this interpretation as there is continuing debate in the literature as to the latent structure of the HADS and its utility in differentiating and detecting clinically relevant levels of anxiety and depression (Coyne & van Sonderen, 2012*a*,*b*; Norton *et al.* 2012). Hence, future studies with a specific focus on identifying the shared and differential cognitive deficits associated with IBS and related psychiatric conditions are needed, with psychiatric status being formally determined using a SCID.

Of note, patients with CD exhibited a reduction in their capacity to cope with Stroop interference when compared to healthy control participants. They did not, however, exhibit visuospatial memory impairment. This suggests a disorder-specific mechanism by which HPA-axis dysfunction can impact neurobiologically on cognitive performance. Cognitive function in IBD has not been well characterized, with only one study in the literature, lacking a healthy control comparison group, reporting a beneficial outcome of iron replacement therapy on cognitive performance in anaemic patients with CD (Wells *et al.* 2006). However, brain morphological abnormalities have recently been reported in CD (Agostini *et al.* 2013) and a subsequent review article has proposed that CD may be associated with altered cognitive performance due to these abnormalities (Vogt, 2013). Our results lend substantial support to this hypothesis and indicate that further investigations are needed at a structural and functional brain-imaging level to definitively link poor cognitive performance to the proposed morphological brain changes in CD.

A limitation of our study is the heterogeneous nature of our IBS population. There is some evidence to suggest that psychiatric diagnoses and possibly central functioning varies between IBS-D, IBS-C and IBS-A subtypes (Tillisch *et al.* 2005). Future studies, adequately powered to detect subgroup differences of cognitive performance in IBS, are needed, in addition to detecting potential differences in PAL performance between patients with CD and controls and patients with CD and IBS. In addition, the inclusion of patients with active and inactive CD and ulcerative colitis, in conjunction with a more temporally specific measure of GI symptoms than the HBI, will be important in identifying whether cognitive differences exist between the two categories of IBD, due to disease activity and general GI symptomatology. Our results suggest that CD patients exhibit some differences in cognitive performance on the PAL and future studies should aim to explore this possibility in more detail. Whether the cognitive alterations identified here are state or trait cognitive markers requires further investigation by prospective and longitudinal study designs, particularly in relation to the potential impact of fluctuating symptoms and their severity on cognitive performance in IBS.

Our groups differed with regard to gender and it is important to note that gender differences in brain responses to visceral pain stimulation have been observed in IBS (Berman *et al.* 2000; Naliboff *et al.* 2003). Although small, and notably inconsistent, gender differences on visual–spatial tasks such as tests of spatial orientation or verbal abilities in healthy populations are commonly reported in the literature (Weiss *et al.* 2003), the influence of gender on visuospatial episodic memory performance in the PAL, to the best of our knowledge, has not been specifically investigated. It is therefore difficult to know whether gender differences impacted on our results, and future studies should aim to delineate the effects of gender on performance on the PAL and other assessments in IBS. Such studies will also need to examine the role that important factors such as age and menstrual cycle may play in cognitive performance in IBS.

In conclusion, IBS patients in the current study displayed a subtle but significant deficit on a hippocampal-mediated test of visuospatial episodic memory, which was related to morning cortisol levels and independent of psychiatric co-morbidity. Our results must be considered preliminary and this study as exploratory, and there is a clear need for follow-up studies to confirm these findings and to address the limitations discussed. Nonetheless, when considering that IBS is a common disorder and typically emerges in young adults who are involved in formal education or are in an early phase of career development, assessing the impact of IBS on cognition is of paramount importance. Daily stressors play a key role in exacerbating symptoms in IBS (Levy *et al.* 1997; Bennett *et al.* 1998) and our results suggest that, from a clinical point of view, therapies aimed at reducing stress or normalizing HPA-axis functioning should be considered a key line of treatment to reduce not only the impact on GI symptoms in IBS but also the central impact on cognitive function. Our results also have important implications for individuals who suffer from CD, which need to be addressed. Finally, in keeping with our primary hypothesis, these data support the view that IBS is a disorder associated with cognitive impairment (Kennedy *et al.* 2012) and warrant further investigation to identify the neurobiological mechanisms influencing cognitive performance in this debilitating disorder of the brain–gut axis.
